# F160. CLASSIFYING SCHIZOPHRENIA BY PATTERNS OF BOLD FLUCTUATIONS USING MULTIVARIATE PATTERN RECOGNITION ANALYSIS

**DOI:** 10.1093/schbul/sby017.691

**Published:** 2018-04-01

**Authors:** Jing Shang, Christian Sorg, Josef G Bäuml, Joseph Kambeitz, Felix Brandl, Nikolaos Koutsouleris

**Affiliations:** LMU

## Abstract

**Background:**

Schizophrenia is characterized by changes in both ongoing blood oxygenation level dependent (BOLD) signal fluctuations of resting-state fMRI and their coherence in terms of functional connectivity. The current study asks the question whether individualized patterns of BOLD fluctuations are able to classify schizophrenia patients from healthy controls.

**Methods:**

To investigate this question, 61 schizophrenia (SZ) patients and 73 healthy controls (HC) were obtained from a Mind Research Network COBRE dataset available via COINS (http://coins.mrn.org/dx). The amplitude of low-frequency fluctuations (ALFF) was used as main outcome measure reflecting BOLD fluctuations. Multivariate pattern classification framework based on support-vector machines (SVM) was used to generate and validate ALFF patterns for group separation.

**Results:**

ALFF based classifiers were able to distinguish between SZ patients and HC with 76.9% accuracies (balanced accuracy 76.5%, specificity 80.8%, sensitivity 72.1%, Area Under the Curve: 0.78). Decreased ALFF highly predictive for SZ was located in bilateral somatomotor cortex, cuneus and orbitofrontal cortex. Increased ALFF highly predictive for SZ was located in the thalamus, dorsomedial prefrontal cortex, and precuneus.

**Discussion:**

Conclusions: Our results provide evidence for BOLD-fluctuation pattern could be treated as reliable feature to identify individual patients with schizophrenia from healthy controls. Multivariate pattern analysis such as support vector machine may reliably detect signatures of schizophrenia.

